# Stubborn hypoxemia after mild to moderate sedation and analgesia: A case report

**DOI:** 10.1097/MD.0000000000037059

**Published:** 2024-02-16

**Authors:** Jiayun Zou, Huazhen Wang, Yongshan Nan, Xianglan Jin

**Affiliations:** a Department of Anesthesiology, Yanbian University, Yanbian University Hospital, Yanji, Jilin, P.R. China; b Department of Anesthesiology, Yanbian University Hospital, Yanji, Jilin, P.R. China.

**Keywords:** atelectasis, monitored anesthesia care, stubborn hypoxemia

## Abstract

**Introduction:**

Atelectasis typically denotes the partial or complete collapse of lung segments, lobes, or lobules in individuals, leading to a compromised respiratory function. The prevalence of perioperative atelectasis may be significantly underestimated, particularly among patients subjected to general anesthesia.

**Patient concerns:**

This article conducts a retrospective analysis of a case involving refractory hypoxemia in a patient with a liver tumor who was admitted to Yanbian University Affiliated Hospital (Yanbian Hospital) after undergoing mild-to-moderate sedation and analgesia outside the operating room.

**Diagnosis:**

Based on the results of CT examination and present history, the patient was diagnosed with intraoperative atelectasis.

**Intervention:**

After the surgery, the patient was transferred to the recovery ward, where nasal oxygen therapy and nebulized inhalation treatment were administered. Vital signs were closely monitored at the bedside, gradually returning to the preoperative baseline.

**Outcome:**

Postoperatively, the patient developed atelectasis, with the percentage of lung opacity shown in the image decreasing from 9.2% of the total thoracic cage area to 8.4%.

**Conclusion:**

During non-intubated intravenous anesthesia, patients with compromised pulmonary conditions are more susceptible to refractory hypoxemia. Therefore, a personalized approach should be adopted regarding oxygen concentration and the dosage and type of medication. Additionally, preparations for appropriate airway management measures are essential to safeguard patient safety in the event of respiratory issues.

## 1. Introduction

Atelectasis is usually a loss of ventilation due to atrophy of a segment, lobe, or lobule of a patient’s lung. The incidence of perioperative pulmonary atelectasis may be much higher than one would expect, especially in patients under general anesthesia. One case of intractable hypoxemia in a patient with liver tumor admitted to Yanbian University Hospital (Yanbian Hospital) after mild-moderate sedation and analgesia given during extra operative anesthesia in the operating room is retrospectively analyzed and discussed in the context of related literature.

## 2. Case report

**Figure 1.**
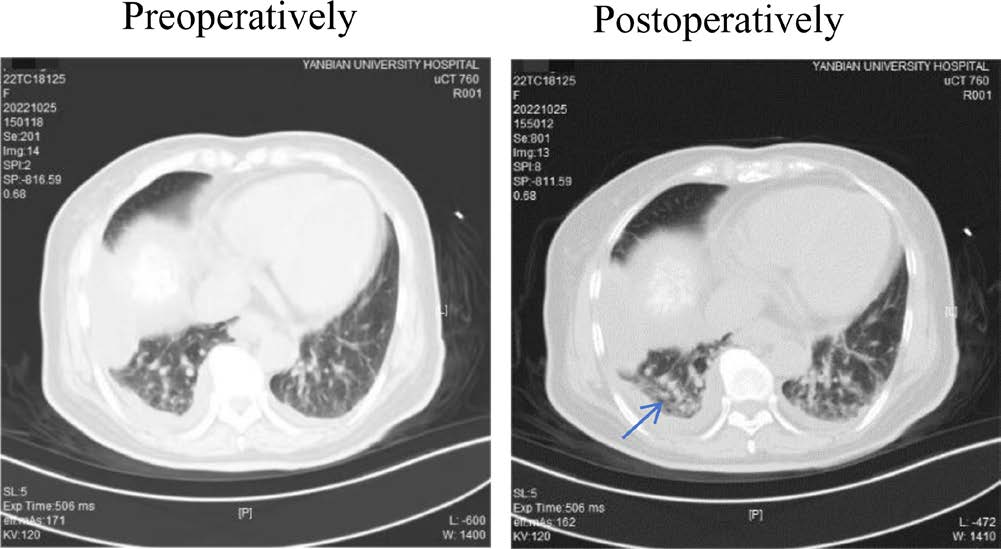
CT images before and after surgery.

We report a case of a female patient who developed pulmonary atelectasis during extra-operative room (CT room) anesthesia. Diagnosis and treatment: a 62-year-old patient was considered to have a malignant tumor in the right lobe of the liver, and after multidisciplinary consultation, surgical treatment or hepatic arterial embolization combined with radiofrequency ablation was recommended. The patient was treated with hepatic tumor arterial embolization on October 20, 2022, and in order to consolidate the treatment, hepatic tumor radiofrequency ablation. Surgical procedure: at 15:00, the operator first located the tumor location under CT guidance in the supine position, at which time lung CT showed chronic inflammation in both lungs (see Fig. 1). The patient was monitored by the anesthesiologist at 15:30, and the blood oxygen was 92%, pulse 90 beats/min, heart rate 90 beats/min, and blood pressure 138/80 mm Hg. The blood oxygen reached 98% with an oxygen flow of 3 L/min and nasal cannula oxygen intake for 5 minutes at 15:40, the anesthesiologist sequentially pushed midazolam injection 1 mg, sufentanil 5 μg, and dexmedetomidine 4 μg, so that the patient entered a light-medium sedation and the Ramsay sedation score was 5. At 15:43, the patient was routinely disinfected, toweled, locally injected with 5% lidocaine 5 mL, and underwent percutaneous liver puncture microwave ablation of liver tumor under ultrasound guidance with a power of 70 W. At 15:45, the blood oxygen saturation gradually decreased to about 91%, and the patient was promptly treated with mandibular support and an oxygen flow of 5 L/min, and the oxygen level was temporarily maintained at about 93%, and the Ramsay sedation score was 4. At 15:48, the patient’s oxygen saturation further decreased to 88%, heart rate was 69, and blood pressure was 155/82 mm Hg. The operator was instructed to stop the operation, wake up the patient immediately, and use ultrasound to preliminarily exclude pneumothorax and continue the operation until the end of the operation at 15:50, and then rowed CT again to assess the ablation range, and the CT of lungs at this time showed that the lower lobe of the right lung was slightly atelectasis and the pleural effusion was obviously increased. As shown in Figure 1, the patient exhibited pulmonary atelectasis and a significant increase in pleural effusion. Naloxone 0.4 mg and flumazenil 0.3 mg were given at the end of the operation, and the patient was instructed to cough strongly several times, but the oxygen saturation was maintained at about 90%. The patient’s condition improved significantly after aggressive postoperative anti-inflammatory and nebulized inhalation treatments.

Nasal catheter oxygen + sedation and analgesia technique is commonly used in the anesthesia of patients with invasive diagnosis and treatment outside the operating room, this patient needs to perform radiofrequency ablation treatment for liver tumor under the combined guidance of CT and ultrasound because of liver cancer, due to simple puncture operation, it has low requirements for muscle relaxation, and it only needs the patient’s cooperation in the state of sedation, and it often adopts local anesthesia + intravenous administration alone to maintain the state of sedation. As we mentioned in the previous article, the incidence of pulmonary atelectasis is very high in the perioperative period, and in the operating room, although we have a lot of monitoring instruments as well as facilities, it is easy to miss the diagnosis, and only in the case of intractable hypoxemia, we can only determine whether there is a possibility of pulmonary atelectasis through anesthesia monitoring and experience. In this case, intractable hypoxemia developed immediately under mild-moderate sedation anesthesia outside the operating room, and coincidentally, in combination with the persistent drop in intraoperative oxygen and CT localization, we also detected pulmonary atelectasis, during which the patient was given nasal catheter oxygen, and ventilation could only be indirectly controlled by controlling the change in the oxygen flow rate to control the concentration of inhaled oxygen.

## 3. Discussion

The occurrence of pulmonary atelectasis is related to many factors, and the known relevant factors are obesity, acute lung inflammation, and advanced age in the preoperative period, and the intraoperative factors are ventilation strategy with high partial pressure of oxygen, ventilation strategy without positive end-expiratory pressure (PEEP) and low tidal volume, and body position, etc.^[1]^ It has been shown in the literature^[2]^ that when maintained in volume-controlled mode with inhaled oxygen concentration of 100%, pulmonary atelectasis develops within 5 minutes, but when the inhaled oxygen concentration is 40% pulmonary atelectasis starts to appear after 40 minutes. This case shows that in patients at high risk for lung inflammation, such as those of advanced age, pulmonary atelectasis occurs earlier and faster than expected, and is difficult to correct.

This case is worth thinking about how to deal with the emergence of pulmonary atelectasis in general anesthesia surgery. Prompt and aggressive management of pulmonary inflammation is essential regardless of the type of anesthesia administered. Intraoperative anesthesiologists usually prevent pulmonary atelectasis through lung-protective ventilation strategies, and in the traditional strategy, half-hourly lung reanimation is usually used to prevent atelectasis, but this case shows us that there is clearly a safety risk for these patients. Therefore, an individualized treatment plan is adopted for each patient, especially in anesthesia for patients who cannot tolerate possible anesthetic complications, a strategy of keeping the lungs open during induction if possible, and advance ultrasound-guided lung recanalization for patients with high risk factors for the possibility of pulmonary atelectasis,^[3]^ among other measures, is taken to ensure the patient’s health, and the inspired oxygen concentration is reduced as much as possible to the lowest acceptable value in the acceptable range during the management period. The lowering of the inhaled oxygen concentration to the lowest acceptable level during management is beneficial in minimizing the development of pulmonary atelectasis.^[4]^

## 4. Conclusion

Oxygen inhalation is necessary during anesthesia, as inhaling pure oxygen can increase the patient’s oxygen reserves to counteract the respiratory challenges induced by surgical stimuli or anesthesia-induced respiratory depression, thus preparing for emergency situations. However, it may also potentially lead to atelectasis. Therefore, precise control of oxygen concentration is crucial.

## Author contributions

**Analysis and interpretation of data, drafting the article for important intellectual content, and final approval:** Xianglan Jin, Yongshan Nan.

**Conception, design, and acquisition of data:** Jiayun Zou, Huazhen Wang.

**Conceptualization:** Jiayun Zou.

**Data curation:** Huazhen Wang.

**Funding acquisition:** Xianglan Jin, Yongshan Nan.

**Investigation:** Jiayun Zou.

**Methodology:** Huazhen Wang.

**Writing – original draft:** Jiayun Zou.

**Writing – review and editing:** Xianglan Jin, Yongshan Nan.
